# Cortical Patterns Shift from Sequence Feature Separation during Planning to Integration during Motor Execution

**DOI:** 10.1523/JNEUROSCI.1628-22.2023

**Published:** 2023-03-08

**Authors:** Rhys Yewbrey, Myrto Mantziara, Katja Kornysheva

**Affiliations:** ^1^Bangor Imaging Unit, Bangor University, Bangor, Wales LL57 2AS, United Kingdom; ^2^Centre for Human Brain Health, School of Psychology, University of Birmingham, Birmingham, B15 2TT, United Kingdom

**Keywords:** fMRI, motor control, MVPA, planning, sequence, timing

## Abstract

Performing sequences of movements from memory and adapting them to changing task demands is a hallmark of skilled human behavior, from handwriting to playing a musical instrument. Prior studies showed a fine-grained tuning of cortical primary motor, premotor, and parietal regions to motor sequences: from the low-level specification of individual movements to high-level sequence features, such as sequence order and timing. However, it is not known how tuning in these regions unfolds dynamically across planning and execution. To address this, we trained 24 healthy right-handed human participants (14 females, 10 males) to produce four five-element finger press sequences with a particular finger order and timing structure in a delayed sequence production paradigm entirely from memory. Local cortical fMRI patterns during preparation and production phases were extracted from separate No-Go and Go trials, respectively, to tease out activity related to these perimovement phases. During sequence planning, premotor and parietal areas increased tuning to movement order or timing, regardless of their combinations. In contrast, patterns reflecting the unique integration of sequence features emerged in these regions during execution only, alongside timing-specific tuning in the ventral premotor, supplementary motor, and superior parietal areas. This was in line with the participants' behavioral transfer of trained timing, but not of order to new sequence feature combinations. Our findings suggest a general informational state shift from high-level feature separation to low-level feature integration within cortical regions for movement execution. Recompiling sequence features trial-by-trial during planning may enable flexible last-minute adjustment before movement initiation.

**SIGNIFICANCE STATEMENT** Musicians and athletes can modify the timing and order of movements in a sequence trial-by-trial, allowing for a vast repertoire of flexible behaviors. How does the brain put together these high-level sequence features into an integrated whole? We found that, trial-by-trial, the control of sequence features undergoes a state shift from separation during planning to integration during execution across a network of motor-related cortical areas. These findings have implications for understanding the hierarchical control of skilled movement sequences, as well as how information in brain areas unfolds across planning and execution.

## Introduction

**Figure 1.**
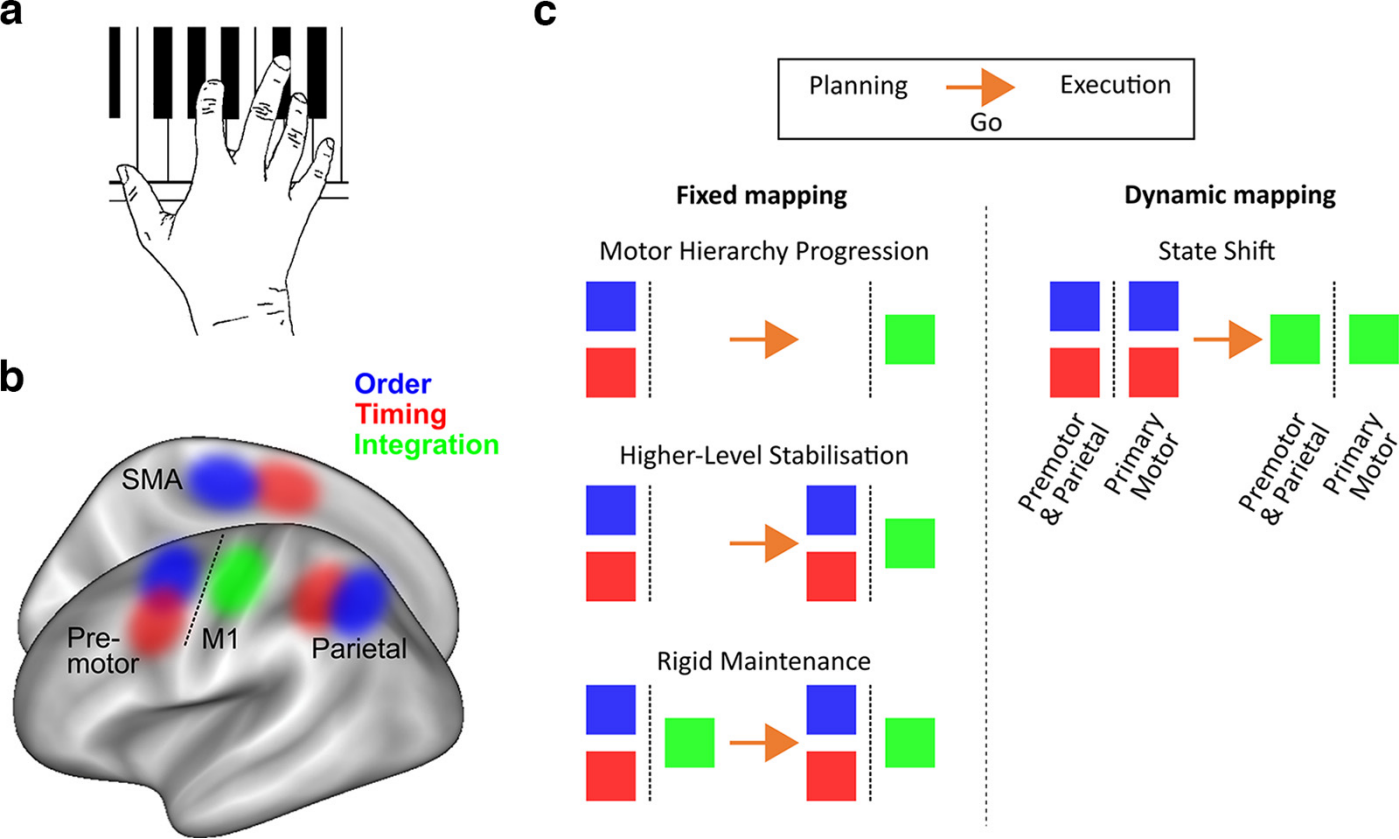
Theoretical framework and hypotheses. ***a***, Skilled sequence production (e.g., when playing a melody on a piano) is characterized by producing movements with a specific order and timing and combining them flexibly trial-by-trial. ***b***, Previous findings localized independent patterns of order and timing to premotor, supplementary motor, and parietal regions, while their integration was found in M1 (Kornysheva and Diedrichsen, 2014). ***c***, How does this mapping evolve across planning and execution? “Fixed mapping” hypotheses state that premotor and parietal regions outside of M1 control order and timing as independent motor sequence features, and M1 itself controls the nonlinear integration of the two during planning and/or production. In contrast, the “Dynamic mapping” hypothesis proposes that there is a state shift within regions from independent feature control during planning to integration during execution (OSF preregistration: https://doi.org/10.17605/OSF.IO/G64HV).

Skilled sequences of movements performed from memory are regarded as a hallmark of human dexterity (Hikosaka et al., 2002; Rosenbaum et al., 2007; Diedrichsen and Kornysheva, 2015). They are essential building blocks of everyday skilled behaviors, from typing, to tying shoelaces, or playing a musical instrument (Fig. 1*a*). In addition to the order of movements in a sequence, the temporal accuracy of the movements can be crucial to the success of the task (e.g., when tapping a Morse code). Previous behavioral (Ullén and Bengtsson, 2003; Gobel et al., 2011; Kornysheva et al., 2013), computational (Zeid and Bullock, 2019; Calderon et al., 2022), neurophysiological (Merchant et al., 2013; Zimnik and Churchland, 2021; Lafuente et al., 2022), and neuroimaging findings (Bengtsson et al., 2004; Kornysheva and Diedrichsen, 2014; Kornysheva et al., 2019) established that movement order is controlled independently of timing, and vice versa, whenever motor sequences incorporated temporally discrete subgoals. This includes sequences that are extensively trained and performed from memory without external guidance, characteristic of motor sequence execution in the real world. The integration of movement timing and order has been studied in the context of execution (Shin and Ivry, 2002; Kennerley et al., 2004; O'Reilly et al., 2008; Kornysheva et al., 2013; Kornysheva and Diedrichsen, 2014), but we currently do not know whether the binding of order and timing takes place before the initiation of the first movement, and which motor-related cortical areas underlie this process.

Neural and hemodynamic activity patterns in contralateral primary motor (M1) and sensorimotor (S1), premotor, and parietal cortices show informational tuning to trained motor sequences (Tanji and Shima, 1994; Matsuzaka et al., 2007; Wymbs et al., 2012; Picard et al., 2013; Wiestler and Diedrichsen, 2013; Kornysheva and Diedrichsen, 2014; Wiestler et al., 2014; Wymbs and Grafton, 2015; Yokoi et al., 2018; Yokoi and Diedrichsen, 2019; Berlot et al., 2020). Specifically, activity patterns outside the primary motor cortex (premotor, supplementary motor, and parietal areas) contain high-level information (e.g., about sequence chunks), positional rank in the sequence (Tanji and Shima, 1994; Yokoi and Diedrichsen, 2019; Russo et al., 2020), and spatial, rather than body-centered, coordinates (Wiestler et al., 2014). Further, activity patterns in these regions can generalize across different pairings of movement order and timing (Kornysheva and Diedrichsen, 2014) (Fig. 1*b*). In contrast, activity patterns in contralateral M1/S1 are associated with the planning and execution of single movements in a sequence (Yokoi et al., 2018; Berlot et al., 2020; Zimnik and Churchland, 2021; Ariani et al., 2022), body-centered coordinates (Wiestler et al., 2014), and information about unique sequence order and timing integration, suggesting lower-level representations driven by motor implementation (Kornysheva and Diedrichsen, 2014) (Fig. 1*b*).

Despite the progress made, it remains uncertain when and where motor-related cortical areas integrate the order and the timing of movements trial-by-trial. One possibility is that premotor and parietal regions show a fixed mapping to high-level independent, and M1/S1 to lower-level integrated sequence features, respectively (Kornysheva and Diedrichsen, 2014). These may be activated simultaneously or sequentially depending on the perimovement phase, but their informational content could remain stable (Fig. 1*c*, “Fixed mapping”). Alternatively, the tuning to high- and low-level features may change dynamically with phase with the same regions parsing sequence order and timing during planning but integrating these sequence features during execution (“Dynamic mapping”).

We trained participants to produce five-element finger press sequences comprised of two finger orders and two temporal interval orders (timings) from memory in a delayed production paradigm. To disentangle planning from execution using fMRI, activity was extracted from No-Go and Go trials, respectively. We utilized multivariate pattern analysis (MVPA) to decode fMRI patterns related to the planned and executed sequence order and timing. Our results provide strong evidence for the integration of sequence order and timing during sequence execution only, but not during planning. Further, they support the idea that contralateral cortical regions are not fixed in their informational content but update their tuning dynamically.

## Materials and Methods

### Participants

Twenty-four neurologically healthy participants, 14 females and 10 males (mean = 21.00 years, SD = 1.64 years), met all behavioral and imaging requirements after completing the 3 d experiment. Twenty-three participants were right-handed with a mean Edinburgh Handedness Inventory (https://www.brainmapping.org/shared/Edinburgh.php) (adapted from Oldfield, 1971) score of 75.22 (SD = 20.97, range: 25-100), one was left-handed with an Edinburgh Handedness Inventory score of −70. Although our preregistration (www.osf.io/g64hv) stated we would exclude left-handed individuals, we included this participant as their data were not qualitatively different from the rest of the sample. Data were collected from an additional 17 participants but were excluded. One participant was excluded because of unforeseen technical difficulties with the apparatus, and one participant was excluded because of a corrupted functional scan. Fifteen further participants did not reach target performance after 2 d of training. Target performance consisted of an error rate <20% (mean = 6.54%, SD = 6.03%, for the group) and distinct sequence timing structures that transferred across sequence finger orders (see Results). Participants were recruited either through social media and given monetary reward at a standard rate, or through a participation panel at Bangor University and awarded module credits for their participation. Participants with professional musical qualifications were excluded from recruitment. All participants provided informed consent, including consent to data analysis and publication, through an online questionnaire hosted by Qualtrics. This experiment and its procedures were approved by the Bangor University School of Psychology Ethics Committee (Ethics approval number 2019-16478).

### Apparatus

Force data from fingers of both the right and left hands were recorded at a sample rate of 1000 Hz using two custom-built force transducer keyboards (10 channels). Each key had a groove within which the respective fingertip was positioned. A force transducer (Honeywell FS Series, with a range of up to 15 N) was located under each groove and recorded the respective finger force without crosstalk between channels. Force data acquisition occurred in each trial from 500 ms before sequence cue onset to the end of the production period in production trials, and the end of the false production period in No-Go trials. The keys could be adjusted in position by sliding them up and down individually along the keyboard plane to achieve the most comfortable position for the hand and wrist when seated during training or in supine position in the MRI scanner, respectively. Once adjusted, the position of the keys was fixed. Traces from the right hand were baseline-corrected by the first 500 ms of acquisition (500 ms before the sequence cue) and smoothed to a Gaussian window of 100 ms, trial-by-trial. Button presses were defined as the point at which forces above baseline exceeded a fixed threshold (2.5 N for the first 8 participants and 1 N for the subsequent 16 of 24 participants). Press timings were identified by the timestamp provided by National Instruments Data Acquisition Software (National Instruments) associated with the data point at which the respective threshold was exceeded.

During behavioral training sessions, participants were seated at a wooden table ∼75 cm away from a 19 inch LCD LG Flatron L1953HR, at a resolution of 1280 × 1024, at a refresh rate of 60 Hz. Their hands were occluded by a horizontally positioned panel on posts around the force boxes. During fMRI sessions, stimuli were presented on an MR Safe BOLDScreen 24 inch, at a resolution of 1920 × 1200 and a refresh rate of 60 Hz. Participants laid supine on the scanner bed, and the two force transducers were positioned on a plastic support board resting on their bent upper legs to enable comfortable and stable positioning of the hands.

### Behavioral task

Participants were trained to produce four five-finger sequences with defined interpress intervals (IPIs) from memory in a delayed sequence production paradigm. Go trials began with a fractal image (Sequence cue) presented for 400 ms, which was associated with a sequence. The mapping between fractal image and each sequence was defined randomly for each participant. Following the Sequence cue, a fixation cross was shown to allow participants to prepare the upcoming sequence; display length of this fixation cross was jittered at durations of 600, 1100, 1600, and 2100 ms, pseudorandomized across trials within blocks. A black hand with a green background (Go cue) then appeared for 4000 ms to cue sequence production. Succeeding the Go cue, another fixation cross was presented in a jittered fashion at durations of 500, 1000, 1500, and 2000 ms. Feedback (for more details, see Feedback) was then presented to participants for 1000 ms, followed by a jittered intertrial interval (ITI) duration of 1000, 1500, 2000, and 2500 ms. Visually guided (Instructed) Go trials during training were presented in the same fashion, albeit featuring a Go cue with a gray background, and a red dot on the tip of each finger on the hand image would move from finger to finger in the target production order and in-pace with the target timing structure. No-Go had the same structure to Go trials, but No-Go cue was shown succeeding the preparatory fixation cross. Instead of the Go cue, the fixation cross continued to show for an additional 1000 ms. As in Go trials, this phase of the trial was followed by a fixation cross, feedback, and ITI.

Four target sequences consisted of permutations of two finger orders (Order 1 and 2) and two IPI orders (Timing 1 and 2) matched in finger occurrence and sequence duration. Sequence orders were generated randomly for each participant. All trained sequences began with the same finger press to avoid differences in the first press driving the decoding of sequence identity during preparation (Yokoi et al., 2018). Ascending and descending press triplets and any identical sequences were excluded. Timing structures were the same across participants, to allow for comparison of timing performance across participants. The two trained timing structures consisted of four target IPI sequences as follows: 1200 ms-810 ms-350 ms-650 ms (Timing 1), and 350 ms-1200 ms-650 ms-810 ms (Timing 2). To assess whether participants maintained the target timing structure despite individual tendencies to lengthen or compress overall sequence length, we calculated timing error for each participant relative to their average total production length. This was calculated offline by normalizing target and produced IPIs as a percentage of the participant's average total sequence length during the session across sequences, then calculating the cumulative percent deviation from target for each IPI, averaged across trials.

Feedback was given to participants trial-by-trial on a points-based scale ranging from 0 to 10. Points were based on initiation reaction time and temporal deviation from target timing calculated as a percentage of the target interval length. For initiation reaction time, up to 5 points were awarded for a fast initiation reaction time as follows: 5 points for presses within 200 ms of the Go cue, 4 points for presses within 200-360 ms, 3 points for presses within 360-480 ms, 2 points for presses within 480-560 ms, 1 point for presses within 560-600 ms, and 0 points for presses >600 ms. For IPI performance, up to 5 points were awarded based on deviation from target IPI structure in percent of respective interval to account for the scaling of temporal error with IPI length (Rakitin et al., 1998). Five points were awarded for average deviations of IPIs from target for each trial which was lower than 10%, 4 points for 10%-20%, 3 points for 20%-30%, 2 points for 30%-40%, 1 point for 40%-50%, and 0 points for >50%. If the executed press order was incorrect, participants were awarded 0 points for the trial. If the executed press order was correct, they were awarded their earned timing points. To discourage premature key presses in the preparation period of Go trials and No-Go trials, 0 points were awarded if participants exceeded a force threshold during preparation above the baseline period. In No-Go trials, 5 points were awarded if no press was made as instructed. A monetary reward of £10 was offered to the 2 participants who accumulated the most points across the course of the experiment, to incentivize good performance.

Participants were presented with a feedback screen after each trial showing the number of points achieved in the current trial, as well as feedback on whether they pressed the correct finger at the correct time. Total points accumulated across the whole experiment were shown at the end of each block. A horizontal line was placed in the center of the screen, with four symbols displayed equidistantly along the line which represented each of the five finger presses. An “X” indicated a correct finger press, and a “–” indicated an incorrect finger press for each sequence position. The vertical position of these symbols above (“too late”) or below (“too early”) the line was proportional to the participant's timing of the respective press relative to target IPI (in %). Using these cues, participants could adjust their performance online to ensure maximum accuracy of sequence production and prevent a drift in performance from memory following training. During the first 2 d of training, auditory feedback in the form of successive rising tones corresponding to the number of points (0-10) was played alongside the visual feedback. Auditory feedback was absent during the fMRI session, to prevent any auditory processing driving decoding accuracy.

### Procedure

**Figure 2.**
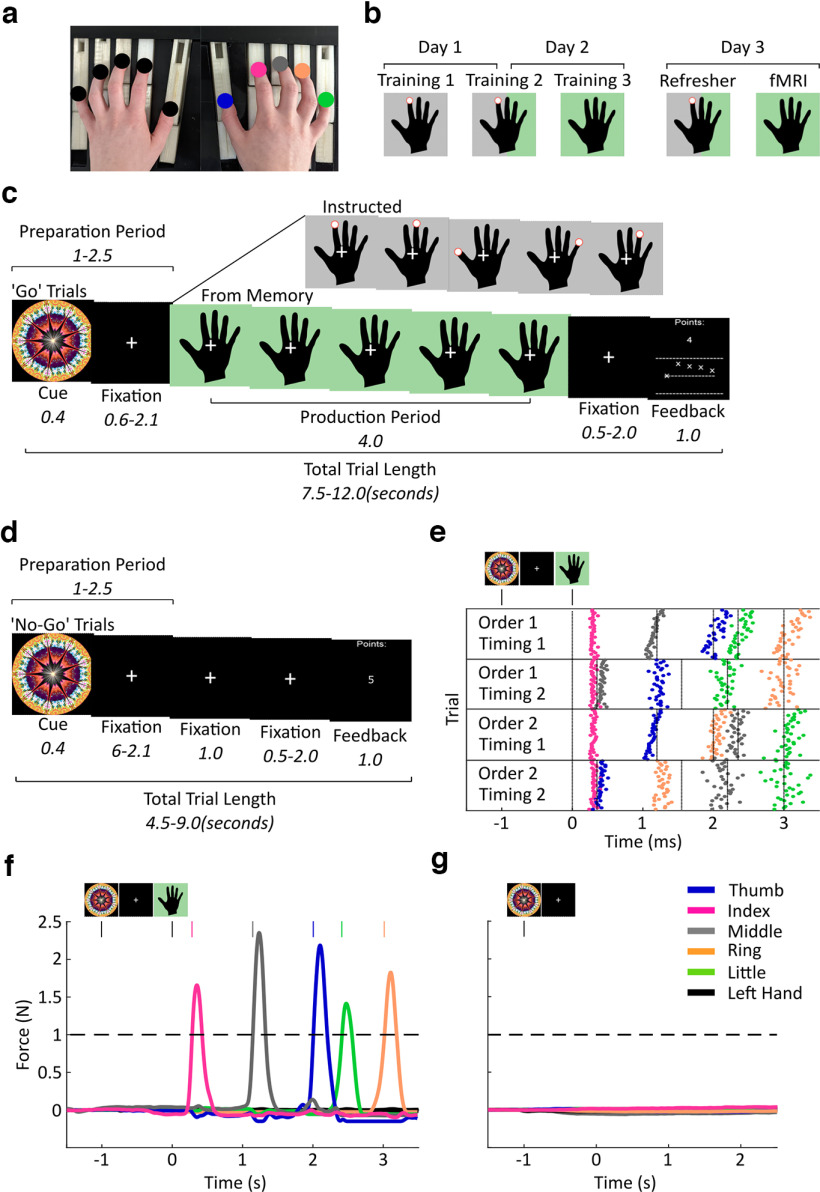
Experimental and trial designs. ***a***, Participants produced finger presses on a 10-finger force transducer keyboard. The hands were visually occluded from the participants' view by a panel during training and when lying in a supine position during the fMRI session. Target fingers on the right hand are indicated by different colors that also correspond to the legend in later panels. Fingers on the left (inactive) hand are marked as black. ***b***, Trial type proportions on each experimental day progressed from 100% instructed (Training 1) to 50%/50% mixed (Training 2) to 100% from memory (Training 3) trials during the last stage of training and during fMRI. For a detailed overview of trial numbers during each session, see Table 1. Black hands with a gray background and a red finger cue represent visually instructed trials. Black hands with a green background represent trials with sequence production from memory. ***c***, Go trials from memory consisted of a Sequence cue, followed by a fixation cross and a Go cue instructing a production period. The occurrence of the Go cue was the onset of the respective hand stimulus. The trial ended with a feedback screen which indicated finger and temporal accuracy relative to a target sequence. Instructed trials, shown as an inset at the top of the image, followed the same trial structure as from memory trials, but displayed visual finger cues to aid production. ***d***, No-Go trials consisted of a Sequence cue, followed by a fixation cross without a Go cue, and feedback screen. ***e***, A raster plot shows all button press timings in correct trials produced from memory across the entire fMRI session in one representative participant. Horizontal lines separate the different sequences that followed a two finger order by two timing design (for details, see Materials and Methods). Vertical dotted lines indicate target press timings. Each colored dot represents a different effector, see corresponding legend. ***f***, Example force traces from 10 channels corresponding to the fingers on the right (colored) and left hands (black) in one representative Go trial during fMRI. Horizontal dashed line indicates the finger press threshold. Colored vertical lines indicate the time point at which a press was detected from the respective finger. ***g***, Example force traces, as in ***f***, from one representative No-Go trial.

Training duration was fixed across participants and occurred across the first 2 d of the experiment over three distinct training stages (for a visual representation of the training stages, see Fig. 2*b*; for trial numbers during each session, see Table 1). In the first training stage, 80% of all trials were instructed Go trials (black hand on gray background, see Fig. 2*c*), and the remaining 20% were No-Go trials. During the second training stage, 40% of trials were instructed Go trials, 40% were from-memory Go trials (black hand on green background, see Fig. 2*c*), and 20% were No-Go trials (see Fig. 2*d*). In the third and final stage of training, 80% of trials were from-memory Go trials, and 20% were No-Go trials. Each stage of training consisted of 240 trials for a total of 720 trials across all three training sections. The third and final day consisted of a short refresher stage of 40 trials, made up of the same proportion of trials as the second stage of training, during which T1 images were collected. Following this refresher stage, there was an fMRI stage consisting of 288 trials (48 trials in each block) featuring 50% from-memory Go trials and 50% No-Go trials.

**Table 1.** Distribution of trial types across experimental phases*^a^*

	Day 1				Day 2			Day 3	
	Example	Pre-training test	Training 1	Training 2	Training 2	Training 3	Post-training test	Refresher	Test (fMRI)
Instructed trials	4 (33%)	32 (100%)	16 (80%)	8 (40%)	8 (40%)	0	32 (100%)	8 (40%)	0
Memory trials	4 (33%)	0	0	8 (40%)	8 (40%)	16 (80%)	0	8 (40%)	24 (50%)
No-Go trials	4 (33%)	0	4 (20%)	4 (20%)	4 (20%)	4 (20%)	0	4 (20%)	24 (50%)
Total trials per block	12	32	20	20	20	20	32	20	48
Number of blocks	1	4	12	6	6	12	4	2	6
Repetitions per condition per block	2	8	4	4	4	4	8	4	6

*^a^*The first half of Training 2 occurred on day 1, the second half on day 2.

**Figure 3.**
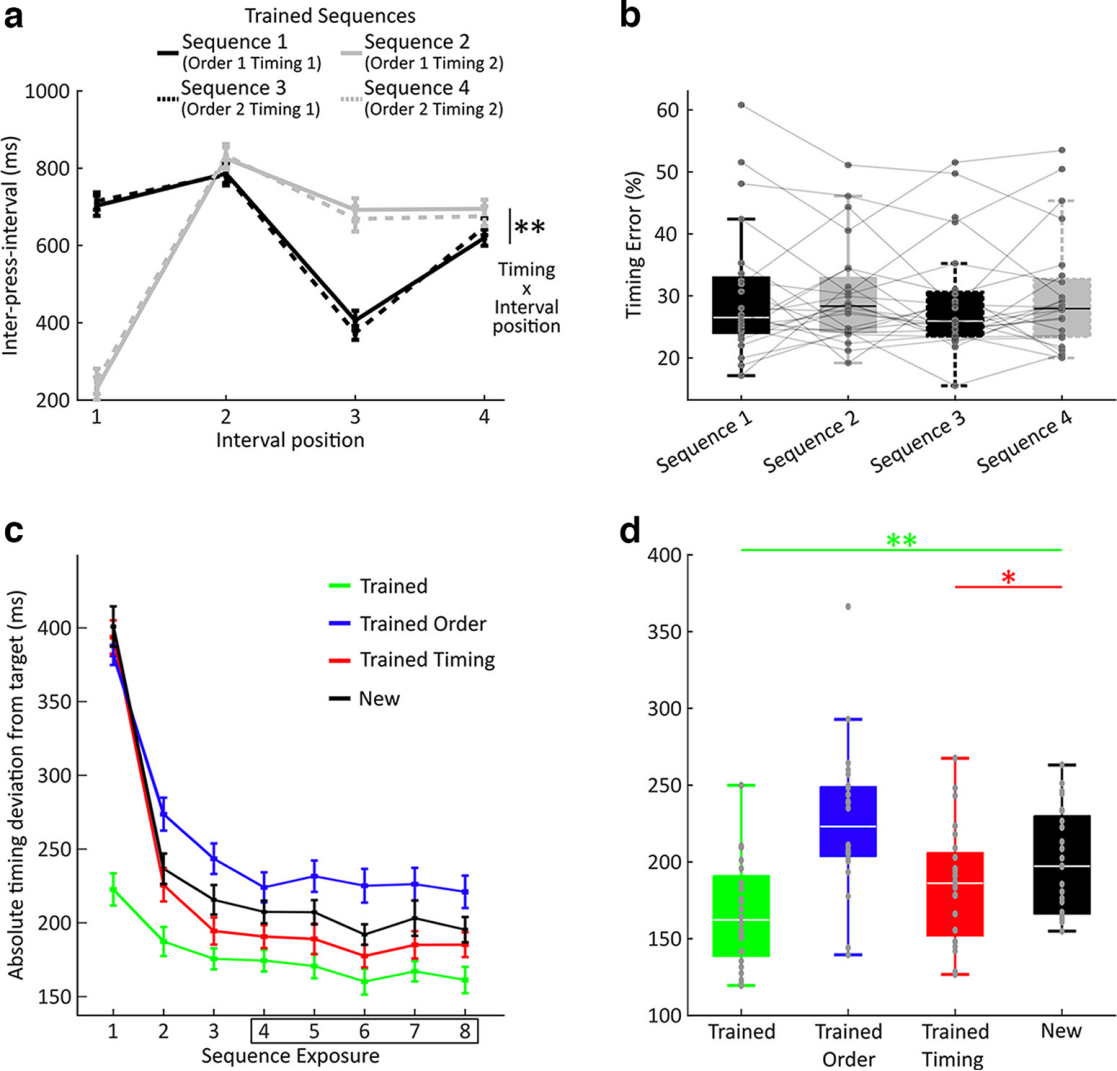
Sequence timing and feature transfer. ***a***, IPI structure of the four trained sequences during the fMRI session (day 3). An interaction between sequence timing and interval position shows distinct IPI sequences between Timing 1 and Timing 2 across finger order sequences Order 1 and 2. **p < .01; repeated measures ANOVA. ***b***, Timing error (normalized to the target interval durations, see Materials and Methods) during the fMRI session did not differ between sequences. ***c***, Behavioral transfer results from the synchronization task obtained from instructed Go cue trials following the last training stage on day 2. Absolute deviation from target timing is shown across sequence repetitions for trained sequences (green), sequences with trained finger orders, but unfamiliar timing (blue), sequences with trained timing, but unfamiliar order (red), and new sequences with both unfamiliar finger order and timing (black). ***d***, Absolute deviation from target timing, as in ***a***, extracted from the fourth to the last sequence repetition as in previous work (e.g., Kornysheva et al., 2019). Significance of *t* tests to identify performance benefits compared with new sequences is shown by colored asterisks and horizontal lines. The trained order condition showed an increase in synchronization error (*p* = 0.002, two-tailed *t* test), suggesting interference rather than benefits related to sequence feature transfer. **p < 0.01; *p < 0.05; one-sided *t* test. The boxplots indicate the median as a white line with the box size delineating the 25th to 75th percentiles, respectively. Lower and upper whiskers correspond the minimum and maximum values, respectively. Outliers are drawn as points.

In addition, before and after the last training stage, participants completed a synchronization task during which they were asked to synchronize their respective presses to a visual finger cue, as in the first stages of training consisting of four blocks of 32 trials, which included trained sequences, sequences with new timings but the same orders (order transfer), sequences with the same timings but new orders (timing transfer), and new sequences. Trial structure was identical to instructed Go trials. There were four sequences belonging to each condition, and each sequence was shown for eight consecutive exposures (see Fig. 3*c*) to assess short-term learning gains. We expected that participants would show more accurate synchronization to visual sequences when they encountered trained sequences as well as sequences with a trained finger order or trained timing compared with untrained control sequences following the completion of training.

### MRI acquisition

Images were obtained on a Philips Ingenia Elition X 3T MRI scanner using a 32-channel head coil. T1 anatomic scans were acquired using a MPRAGE scan at a 0.937 × 0.937 × 1 resolution, with an FOV of 240 × 240 × 175 (A-P, R-L, F-H), encoded in the anterior-posterior dimension.

T2*-weighted functional images were collected across six runs of 230 volumes each with a TR of 2 s, a TE of 35 ms, and a flip angle of 90°. The voxel size was 2 mm isotropic, at a slice thickness of 2 mm, with 60 slices. These were obtained in an interleaved odd-even EPI acquisition at a multiband factor of 2. Four images were discarded at the beginning of each run to allow the stabilization of the magnetic field. The central PFC, the anterior temporal lobe, and ventral parts of the cerebellum were not covered in each participant. Jitters were used within each trial during preparation periods, post-production fixation crosses, and ITIs, to vary which part of the trial is sampled by each TR and therefore give us a more accurate estimate of the Hemodynamic Response Function (HRF) (Serences, 2004).

### Preprocessing and first-level analysis

All fMRI preprocessing was completed using SPM12 (revision 7219) on MATLAB (The MathWorks). Slice timing correction was applied using the first slice as a reference to interpolate all other slices to, ensuring analysis occurred on slices which represent the same time point. Realignment and unwarping were conducted using a weighted least-squares method correcting for head movements using a 6-parameter motion algorithm. A mean EPI was produced using SPM's Imcalc function, wherein data acquired across all six runs were combined into a mean EPI image to be coregistered to the anatomic image. Mean EPIs were coregistered to anatomic images using SPM's coreg function, and their alignment was checked and adjusted by hand to improve the alignment, if necessary. All EPI runs were then coregistered to the mean EPI image.

For the GLM, regressors were defined for each sequence separately for both preparation and production. Preparation- and production-related BOLD responses were independently modeled from No-Go and Go trials, respectively, to tease out activity from these brief trial phases despite the hemodynamic response lag (Logothetis, 2003). The preparation regressor consisted of boxcar function starting at the moment of the Sequence cue in No-Go trials and lasting for the duration of the maximum possible preparation phase (2500 ms). The production regressor consisted of a boxcar function starting at the onset of the first press with a fixed duration of 0 (constant impulse), to capture activity related to sequence initiation and extract sequence production-related activity from the first finger press that was matched across sequences within each participant. We aimed at capturing BOLD responses related to neuronal populations that become differentially active for different sequences (Tanji and Shima, 1994), for which a single estimate of sequence production has been used to successfully identify sequence representations in a number of previous fMRI studies (Wiestler and Diedrichsen, 2013; Kornysheva and Diedrichsen, 2014; Nambu et al., 2015; Yokoi et al., 2018; Berlot et al., 2020). We used a separate pilot dataset (*N* = 9) recorded before the preregistration of the study to determine the optimal GLM regressor model for the execution period. To be certain that the constant impulse model provided the best model for sequence production, we assessed contrast values extracted from a spherical Region Of Interest (ROI) centered on M1a (MNI coordinates: [−38, −31, 48]) with a radius of 6 mm obtained from a separate pilot dataset (*N* = 9) for a model containing variable epoch versus constant impulse regressor for sequence execution. A repeated-measures *t* test found that the constant impulse GLM produced significantly higher contrast values (mean = 10.89, SD = 3.07) than the variable epoch GLM (mean = 3.64, SD = 1.27; *t*_(8)_ = 10.24, *p* < 0.001, *d* = 3.41). This may be because of the way that the BOLD response scales nonlinearly with movement initiation rather than movement duration or speed (Khushu et al., 2001).

Additionally, we included regressors of no interest: (1) error trials (incorrect or premature presses during Go trials and presses during No-Go trials), which were modeled from sequence cue onset to the end of the ITI; (2) the preparation period in Go trials (1000-2500 ms from Sequence cue); and (3) the temporal derivate of each regressor. The boxcar model was then convolved with the standard HRF. To remove the influence of movement-related artifacts, we used a weighted least-squares approach (Diedrichsen and Shadmehr, 2005).

### Surface reconstruction

Cortical surface reconstruction was conducted on each participant's T1 anatomic image using Freesurfer's recon-all function (Dale et al., 1999). Surface structures were then coregistered to the symmetrical Freesurfer average atlas (Fischl et al., 1999) using surface Caret (Van Essen et al., 2001). Searchlights for MVPA were then defined on each individual surface using the node maps provided by the surface reconstruction and displayed in atlas space.

### Cross-sectional and ROI analysis

Two cross-sections were defined on the cortical surface: (1) anterior to posterior, running from dorsal premotor cortex (PMd) to occipito-parietal junction and (2) ventral to dorsal, running from ventral premotor cortex (PMv) to supplementary motor area (SMA). These cross-sections were taken from a previous study (Kornysheva and Diedrichsen, 2014). Data points along these axes were extracted to provide a continuous measure along the cortical surface, which was then subjected to a nonparametric permutation analysis to identify clusters which were significantly above baseline (Maris and Oostenveld, 2007). This was conducted as a one-tailed test, with 10,000 permutations, for which Cohen's *d* effect size was calculated by averaging across the values in each significant cluster (Meyer et al., 2021).

ROI analysis was conducted using the Caret toolbox (Van Essen et al., 2001) on ROIs, which were defined based on Caret masks used by several previous studies (Wiestler and Diedrichsen, 2013; Kornysheva and Diedrichsen, 2014; Wiestler et al., 2014; Yokoi et al., 2018), consisting of PMv, PMd, M1, S1, SMA/pre-SMA, anterior superior parietal cortex (SPCa), and posterior superior parietal cortex (SPCp). The preregistration (www.osf.io/g64hv) referred to the SPCa ROI as “SPC” and S1 and SPCp were added after the preregistration. This was to enable comparison to more recent results, including those published after the preregistration, showing S1 (Gale et al., 2021; Ariani et al., 2022) and SPCp involvement in movement planning (Culham and Valyear, 2006; Lindner et al., 2010; Fitzpatrick et al., 2019), as well as to probe the functional differentiation between SPCa and SPCp with respect to sequence representations demonstrated by Yokoi et al. (2018). *Z* values for each classifier were averaged within regions to give an overall value for each decoder. These values were calculated from unsmoothed individual data. One-sample *t* tests against chance level (zero) then identified significantly above-chance decoding values within these ROIs Bonferroni-corrected for six comparisons. To test the hypotheses in Figure 1 (dynamic vs fixed mapping across planning and execution), we performed a repeated-measures ANOVA on decoding values with factors trial phase, region, and classifier.

### MVPA of fMRI

MVPA was conducted using a custom-written MATLAB code to detect sequence-specific representations (Kornysheva and Diedrichsen, 2014; Kornysheva et al., 2019). We used a searchlight of 160 voxels and a maximum searchlight radius of 6 mm. Each searchlight was run on each individual's cortical surface-reconstructed anatomy, projected onto the Freesurfer average atlas (Fischl et al., 1999). The classification accuracy for each searchlight (compare classification procedures below) was assigned to the center of each searchlight. A classification accuracy map was generated by moving the searchlight across the cortical surface (Oosterhof et al., 2011). Mean patterns and common voxel-by-voxel covariance matrices were extracted for each class from training dataset (five of the six imaging runs), and then a Gaussian linear discriminant classifier was used to distinguish between the same classes in a test dataset (the remaining imaging run).

The factorized classification of finger order, timing, and integrated order and timing followed the previous approach (Kornysheva and Diedrichsen, 2014) and performed on betas estimated from the sequence preparation and production periods independently. For the decoding of sequence timing, the classifier was trained to distinguish between two sequences with differing timing but matching order across five runs and was then tested on two sequences with the same two timings paired with a different order in the remaining run. This classification was then cross-validated across runs and across training/test sequences, for a total of 12 cross-validation folds. For the decoding of sequence order, the classifier was trained to distinguish between two sequences of differing orders paired with the same timing and tested on two sequences with the same two orders when paired with a different timing and underwent the same cross-validation procedure. This method of training and testing the linear discriminant classifier allowed for identification of sequence feature representations that were transferrable across conditions they are paired with and therefore independent. The integrated classifier was trained to distinguish between all four sequences on five runs and then tested on the remaining run. Here, the mean activity for each timing (collapsed across two orders) and finger order (collapsed across two timings) condition within each run was subtracted from the overall activity for each run, separately (Kornysheva and Diedrichsen, 2014). This allowed for the measurement of residual activity patterns that were not explained by a linear combination of timing and order. For better comparability across classifiers, the classification accuracies were transformed to *z* scores, assuming a binomial distribution of the number of correct guesses. We then tested these *z* scores against zero (chance level) on cortical cross-sections of interest and in predefined ROIs across participants for statistical analysis. In addition to the main analysis, we provided an exploratory analysis across the whole cortex by performing a random-effects analysis with an uncorrected threshold of *t*_(23)_ > 3.48, *p* < 0.001 and a cluster-wise *p* value for the cluster of that size (Worsley et al., 1996) on the *z*-transformed decoding values for order, timing, and integration. This was Bonferroni-corrected for two hemispheres and the results, including a full table of significant clusters, are available in Table 3.

### Experimental design and statistical analysis

All data collection and analyses were conducted using a repeated-measures design. For the behavioral data, we assessed changes in finger force production from baseline during the preparation period in both Go and No-Go trials using two-tailed paired-samples *t* tests. We also assessed the length of IPIs and timing error during sequence production using a repeated-measures ANOVA with factors timing, order, and interval position. These ANOVAs were conducted both across the group and within each participant to determine effects within individuals. To evaluate accuracy in the synchronization task, we compared absolute deviation from target interval in the trained, order transfer, and timing transfer conditions to the new sequence condition using three one-sided paired-sample *t* tests, in line with previous work (Kornysheva et al., 2013, 2019; Kornysheva and Diedrichsen, 2014).

To investigate fMRI activity increases in motor-related areas during preparation and production, we tested for increases above a baseline along our two cross-sections using a one-tailed nonparametric permutation test with a *p* value threshold of 0.05 and 10,000 permutations (Maris and Oostenveld, 2007). This method was also used to assess *Z*-transformed decoding accuracy above chance in each of our three classifiers. Further, we ran one-sample *t* tests against chance for each classifier within each ROI and trial phase (preparation and production), Bonferroni-corrected for six comparisons (3 classifiers × 2 trial phases). We also ran a repeated-measures ANOVA with factors phase, classifier, and region to assess interaction effects, and ran *post hoc* pairwise comparisons to investigate a significant interaction between phase and classifier. In addition, we investigated percent signal change and decoding accuracy across cortical hemispheres using whole-brain cluster-based analyses, Bonferroni-corrected for two hemispheres (Tables 2 and 3). The significance value was set to *p* = 0.05 with exact *p* values ≥ 0.001 and effect sizes for each test reported throughout. All statistical tests were performed with MATLAB (The MathWorks) and IBM SPSS Statistics 25.0.

**Table 2.** Surface-based clusters with significant % signal change above rest^*a*^

Contrast versus rest	Area (Brodmann area)	Extent	*p* (cluster)	Peak *t*	MNI		
					*x*	*y*	*z*
Preparation	Contralateral						
	Extrastriate visual cortex (BA18)	4886.16	<0.001	7.21	−12	−60	−5
	Pre-SMA (BA32)	2249.6	<0.001	6.99	−11	14	50
	Primary auditory cortex (BA41)	940.58	<0.001	7.84	−44	−29	11
	Posterior cingulate (BA23)	733.45	<0.001	6.86	−11	−38	31
	Anterior insula (BA48)	706.11	<0.001	6.55	−37	−12	3
	Occipitotemporal area (BA37)	698.86	<0.001	6.39	−38	−62	2
	Anterior insula (BA48)	523.72	<0.001	6.67	−46	18	11
	M1 (BA4)	483.3	<0.001	6.86	−46	−10	44
	Inferior parietal (BA39)	480.37	<0.001	5.48	−50	−59	26
	Extrastriate visual cortex (BA18)	477.2	<0.001	6.19	−20	−90	6
	S1 (BA2)	429.79	<0.001	5.06	−18	−39	59
	Orbitofrontal (BA47)	373.43	<0.001	6.82	−23	29	2
	Superior parietal (BA7)	293.49	<0.001	5.52	−21	−49	51
	Posterior cingulate (BA23)	274.22	<0.001	6.71	−13	−55	26
	Pre-SMA (BA32)	231.75	<0.001	5.46	−9	46	6
	Middle temporal (BA21)	179.26	<0.001	6.06	−46	−37	−3
	Middle temporal (BA21)	125.41	<0.001	6.18	−54	−26	−7
	Inferior parietal (BA39)	122.42	<0.001	5.42	−50	−71	16
	Anterior insula (BA48)	115.35	<0.001	4.46	−28	31	29
	Extrastriate visual cortex (BA19)	98.81	<0.001	5.38	−19	−53	3
	Wernicke's area (BA22)	92.06	<0.001	4.82	−53	−19	3
	Occipitotemporal area (BA37)	86.6	<0.001	4.75	−43	−41	−19
	Anterior prefrontal (BA10)	54.95	0.007	4.49	−6	56	13
	Wernicke's area (BA22)	53.73	0.008	4.5	−57	−37	3
	Anterior insula (BA48)	53.07	0.009	4.35	−54	−1	11
	Posterior cingulate (BA23)	51.92	0.01	6.21	−4	−26	38
	Anterior insula (BA48)	51.89	0.01	5.99	−55	3	14
	Primary auditory cortex (BA42)	48.98	0.014	4.71	−57	−37	16
	Superior parietal (BA5)	39.88	0.043	5	−12	−44	45
	Ipsilateral						
	Extrastriate visual cortex (BA18)	2605.25	<0.001	7.71	−14	−73	−8
	Extrastriate visual cortex (BA19)	1523.99	<0.001	7.2	−21	−83	16
	S1 (BA3)	1498.75	<0.001	7.89	−37	−26	37
	Superior parietal (BA5)	1142.07	<0.001	7.88	−17	−53	47
	Occipitotemporal area (BA37)	883.28	<0.001	7.65	−46	−63	−1
	Ventral temporal (BA20)	671.28	<0.001	6.27	−49	−37	14
	Superior parietal (BA40)	336.82	<0.001	5.56	−34	−52	49
	Anterior cingulate (BA24)	282.58	<0.001	5.86	−6	31	12
	Extrastriate visual cortex (BA18)	274.65	<0.001	4.87	−23	−87	3
	Anterior insula (BA48)	268.99	<0.001	6.19	−41	−24	20
	Superior parietal (BA40)	245.49	<0.001	5.49	−31	36	35
	Anterior insula (BA48)	159.52	<0.001	6.36	−55	−8	13
	Pre-SMA (BA32)	145.62	<0.001	5.05	−10	54	22
	Middle temporal (BA21)	122.81	<0.001	4.83	−53	−50	3
	Pre-SMA (BA32)	106.49	<0.001	5.86	−5	44	7
	Anterior cingulate (BA24)	93.31	<0.001	4.94	−9	11	30
	Subgenual area (BA25)	56.97	0.003	5.21	−4	22	6
	Posterior cingulate (BA23)	56.86	0.003	5.13	−4	−12	34
	S1 (BA3)	48.96	0.008	5.22	−54	−17	37
	Middle temporal (BA21)	48.93	0.008	4.64	−49	−34	−4
	Posterior cingulate (BA23)	44.43	0.015	5.35	−4	−30	32
	Pre-SMA (BA32)	43.95	0.016	4.43	−6	48	27
	Dorsolateral prefrontal (BA9)	40.09	0.026	4.57	−12	34	46
	Ectosplenial area (BA26)	36.16	0.045	4.43	−6	−45	25
Production	Contralateral						
	Superior parietal (BA40)	9012.24	<0.001	13.05	−34	−34	38
	Extrastriate visual cortex (BA18)	1166.68	<0.001	7.05	−30	−86	−12
	SMA (BA6)	741.62	<0.001	9.5	−3	−16	59
	M1 (BA4)	717.53	<0.001	8.91	−53	−1	30
	Extrastriate visual cortex (BA18)	701.31	<0.001	6.37	−18	−71	−2
	Extrastriate visual cortex (BA18)	561.59	<0.001	7.03	−20	−86	−21
	Anterior insula (BA48)	400.9	<0.001	6.84	−33	2	7
	Extrastriate visual cortex (BA18)	174.37	<0.001	5.88	−15	−92	−12
	Posterior cingulate (BA23)	103.37	<0.001	5.43	−5	−5	38
	Anterior insula (BA48)	86.36	<0.001	7.69	−26	13	16
	Primary auditory cortex (BA41)	79.07	<0.001	5.6	−48	−44	25
	Broca's area (BA45)	77.51	<0.001	5.8	−45	30	32
	Extrastriate visual cortex (BA18)	45.13	0.001	4.98	−37	−69	−16
	Ipsilateral						
	Anterior insula (BA48)	2828.07	<0.001	9.24	−51	−42	34
	Extrastriate visual cortex (BA19)	2365.33	<0.001	7.46	−46	−73	−21
	Extrastriate visual cortex (BA18)	1295.15	<0.001	6.91	−9	−81	32
	M1 (BA4)	974.65	<0.001	7.87	−55	−4	35
	V1 (BA17)	542.45	<0.001	5.52	−9	−71	5
	SMA (medial BA6)	513.52	<0.001	8.01	−4	−14	61
	PMd (dorsal BA6)	285.07	<0.001	7.48	−29	−7	48
	Extrastriate visual cortex (BA18)	266.94	<0.001	6.91	−21	−89	−13
	M1 (BA4)	239.01	<0.001	8.4	−33	−21	56
	Posterior cingulate (BA23)	159.35	<0.001	6.4	−8	−24	24
	Anterior cingulate (BA24)	70.39	<0.001	5.71	−9	11	31
	Pars opercularis (BA44)	62.97	<0.001	5.49	−48	19	33
	Pars triangularis (BA45)	42.9	0.008	5.16	−43	34	32
	Pars triangularis (BA45)	39.1	0.014	4.87	−38	40	18

*^a^*Results of surface-based random effects analysis (*N* = 24) with an uncorrected threshold of *t*_(23)_ > 3.48, *p* < 0.001. *p* (cluster) is the cluster-wise *p* value for the cluster of that size. The *p* value is corrected over the cortical surface using the area of the cluster (Worsley et al., 1996) and Bonferroni-corrected for two hemispheres. The cluster coordinates reflect the location of the cluster peak in MNI space.

**Table 3.** Surface-based clusters with significant above-chance classification accuracy for the decoding of sequences and their constituent features (order and timing)^*a*^

Classifier	Area (Brodmann atlas)	Extent	*p* (cluster)	Peak *t*	MNI		
					X	Y	Z
Integrated preparation							
Integrated production	Contralateral						
	Superior parietal (BA7)	606.08	<0.001	7.27	−17	−63	56
	Superior parietal (ba5)	183.08	<0.001	6.6	−14	−47	46
	Extrastriate visual cortex (ba19)	62.85	0.008	4.67	−47	−66	−19
	S1 (BA2)	46.26	0.05	4.34	−28	−44	58
	Ipsilateral						
	Inferior parietal (BA39)	69.53	0.004	5.81	−51	−54	29
	Superior parietal (BA7)	63.4	0.008	5.01	−16	−65	49
Order preparation	Contralateral						
	Extrastriate visual cortex (BA18)	61.68	0.012	4.33	−23	−73	−18
	Extrastriate visual cortex (BA18)	56.03	0.022	5.04	−23	−82	−13
	Ipsilateral						
	Extrastriate visual cortex (BA18)	199.36	<0.001	5.56	−9	−80	30
	Extrastriate visual cortex (BA19)	80.09	0.004	4.9	−21	−62	−5
Order production	Contralateral						
	Extrastriate visual cortex (BA18)	118.04	<0.001	6.51	−24	−94	−10
Timing preparation	Ipsilateral						
	Extrastriate visual cortex (BA18)	58.41	0.026	4.69	−19	−81	−22
Timing production	Contralateral						
	SMA (BA6)	130.73	<0.001	5.13	−3	3	53
	Broca's area (BA44)	128.93	<0.001	4.94	−49	9	13
	S1 (BA3)	114.82	<0.001	5.78	−50	−14	39
	Superior parietal (BA40)	76.95	0.01	5.57	−40	−45	36
	Extrastriate visual cortex (BA19)	59.21	0.044	4.36	−35	−81	16
	Inferior parietal (BA39)	57.46	0.05	4.25	−35	−57	28
	Ipsilateral						
	M1 (BA4)	199.04	<0.001	6.08	−52	−11	40
	PMv (ventral BA6)	164.2	<0.001	6.26	−55	7	23
	Inferior parietal (BA39)	155.11	<0.001	5.76	−39	−48	27
	Pars opercularis (BA44)	151.26	<0.001	5.42	−41	22	35
	Inferior parietal (ba39)	93.54	0.002	5.14	−35	−60	28
	Pars triangularis (ba45)	59.82	0.034	4.21	−43	27	17

*^a^*Table of significant surface-based clusters across the cortex (as in Table 2) for the order, timing, and integrated classifiers.

## Results

### Discrete sequence production from memory

Participants were trained to produce four finger-press sequences from memory with the right hand on a force transducer keyboard (Fig. 2*a*). Training consisted of a three-staged transition across 2 d from trials which visually guided sequence production, toward trials which required sequence production entirely from memory (Fig. 2*b*). During fMRI scans taking place on the third day, participants were required to produce movement sequences from memory only (for trial distribution, see Table 1). Sequences were cued 1000-2500 ms before the Go cue by a Sequence cue (abstract fractal image) to prompt the planning of the respective sequence without movement (Fig. 2*c*). To isolate fMRI responses to movement planning without contamination from execution patterns, in addition to Go trials, No-Go trials were implemented which consisted only of the Sequence cue but did not contain a Go cue (Fig. 2*d*). No-Go trials made up 20% of trials during training, and 50% of trials during the fMRI session (see Materials and Methods). Sequence planning during the preparation period in Go and No-Go trials was facilitated through trial-by-trial reward for fast initiation after the Go cue. Fast initiation and accurate sequence performance in Go trials could result in up to double the points of No-Go trials, in line with a previous study (Mantziara et al., 2021). Thus, to achieve fast and accurate performance and maximize the points, it was beneficial to plan the movement in advance of the Go cue. The target sequences were unique combinations of two finger orders consisting of five presses matched in finger press occurrence and two target relative IPI orders involving four IPIs matched in target duration (Fig. 2*e*). The finger orders were generated pseudo-randomly for each participant, but each sequence started with the same finger press within each participant to avoid first-finger identity driving the sequence decoding during the preparatory period (Yokoi et al., 2018). Timing 1 and Timing 2 were the same across participants.

The keyboard recorded isometric force trajectories from fingers of both the active right and the passive left hand concurrently during preparation and production (Fig. 2*f*,*g*). Points were awarded trial-by-trial only if participants did not exceed a force threshold above the baseline period during preparation and No-Go trials. In Go trials, points were calculated based on initiation time after the Go cue, finger press accuracy, and timing accuracy. No-Go trials were rewarded when no responses were made above threshold (see Materials and Methods). To ensure that participants were not prepressing the keys below the force threshold, we checked offline if exerted force of the right hand increased significantly above the baseline level. In No-Go trials, we checked for force increase from the Sequence cue onset to the last possible moment a Go cue could appear if it were a Go trial, to represent the preparatory period. Participants did not increase force during No-Go trials, and instead showed a small but force reduction (mean = 0.154 N, SD = 0.09) relative to baseline (mean = 0.162 N, SD = 0.09; *t*_(23)_ = 3.39, *p* = 0.003, *d* = 0.69). A similar small decrease, rather than an increase, was found in the preparation phase of Go trials (mean = 0.163 N, SD = 0.09) relative to baseline (mean = 0.164 N, SD = 0.09; *t*_(23)_ = 2.44, *p* = 0.023, *d* = 0.50), suggesting that this force decrease associated with planning was not specific to No-Go trials. Importantly, the data show that participants did not engage in any subthreshold prepressing or rehearsal of the sequence during sequence preparation.

All participants that were included in the study following training produced two distinct timing structures across finger orders when performing the sequences from memory during the fMRI session as instructed, resulting in the expected interaction between sequence timing and interval position at the group level (*F*_(1.78,40.77)_ = 73.76, *p* < 0.001, η*p^2^* = 0.762, Greenhouse-Geisser–corrected, repeated-measures ANOVA; Fig. 3*a*). Since trained finger orders were different across participants (see Materials and Methods), we also assessed the main effects of order and timing and their interaction at the individual level. Here 18 of the 24 participants showed a significant order by interval position interaction, and 10 showed a significant three-way interaction between timing, order, and interval position. The presence of these idiosyncratic press timing patterns at the individual level suggests the integration of sequence order and timing features. Crucially, sequence timing error showed no difference between timing structures, suggesting that there were no systematic differences in difficulty for Timing 1 and Timing 2 at the group level (*F*_(1,23)_ = 0.07, *p* = 0.792, η*p^2^* = 0.003; Fig. 3*b*). At the individual level, 10 participants showed a significant main effect of order, 15 showed a significant main effect of timing, and 4 showed a significant interaction between order and timing, again suggesting an integration of the two sequence features.

Learning new sequences is facilitated if the order or the timing of the sequence has been previously trained (Shin and Ivry, 2002; Ullén and Bengtsson, 2003; O'Reilly et al., 2008; Kornysheva et al., 2013, 2019; Kornysheva and Diedrichsen, 2014). Behavioral transfer to new sequences can be taken as evidence for independent control of sequence order and timing, respectively. Accordingly, we set out to measure behavioral transfer following training. Participants completed a post-training test on day 2 involving a synchronization task which assessed how well participants could synchronize to a visually guided sequence. The trials in each condition were presented in a blocked manner with eight repetitions to assess short-term learning gains related to trained finger order and timing (Fig. 3*c*) analogous to previous studies (Kornysheva et al., 2013, 2019; Kornysheva and Diedrichsen, 2014). Since the transfer of trained sequence timing to a new finger order only takes place after three exposures, synchronization performance was only assessed from the fourth sequence exposure onwards consistent with previously reported analyses (Kornysheva et al., 2013, 2019; Kornysheva and Diedrichsen, 2014). We compared each condition (trained, order transfer, timing transfer) to new sequences (mean = 196.15 ms, SD = 34.00 ms) in a one-tailed paired-sample *t* test. As expected, trained sequences (mean = 160.43 ms, SD = 33.09 ms) showed a performance advantage (*t*_(23)_ = 6.34, *p* > 0.001, *d* = 1.29), and so did sequences with trained timing and a new order (mean = 182.10, SD = 39.72; *t*_(23)_ = 2.09, *p* = 0.024, *d* = 0.43), replicating previous findings (Kornysheva et al., 2013, 2019; Kornysheva and Diedrichsen, 2014) (Fig. 3*d*). In contrast to earlier reports, order transfer sequences (mean = 223.87, SD = 48.30) showed a worse performance relative to a new sequence (*t*_(23)_ = 3.52, *p* = 0.002, *d* = 0.72, two-tailed test). While knowledge of both features of a sequence combined, or just its timing, facilitated task performance, knowledge of sequence order hindered future learning of novel sequence acquisition when paired with a new timing structure. This implies that the participants in our study acquired a stronger independent representation of timing than of finger order which was closely integrated with a particular timing structure during production.

### Activity increases during preparation and production

**Figure 4.**
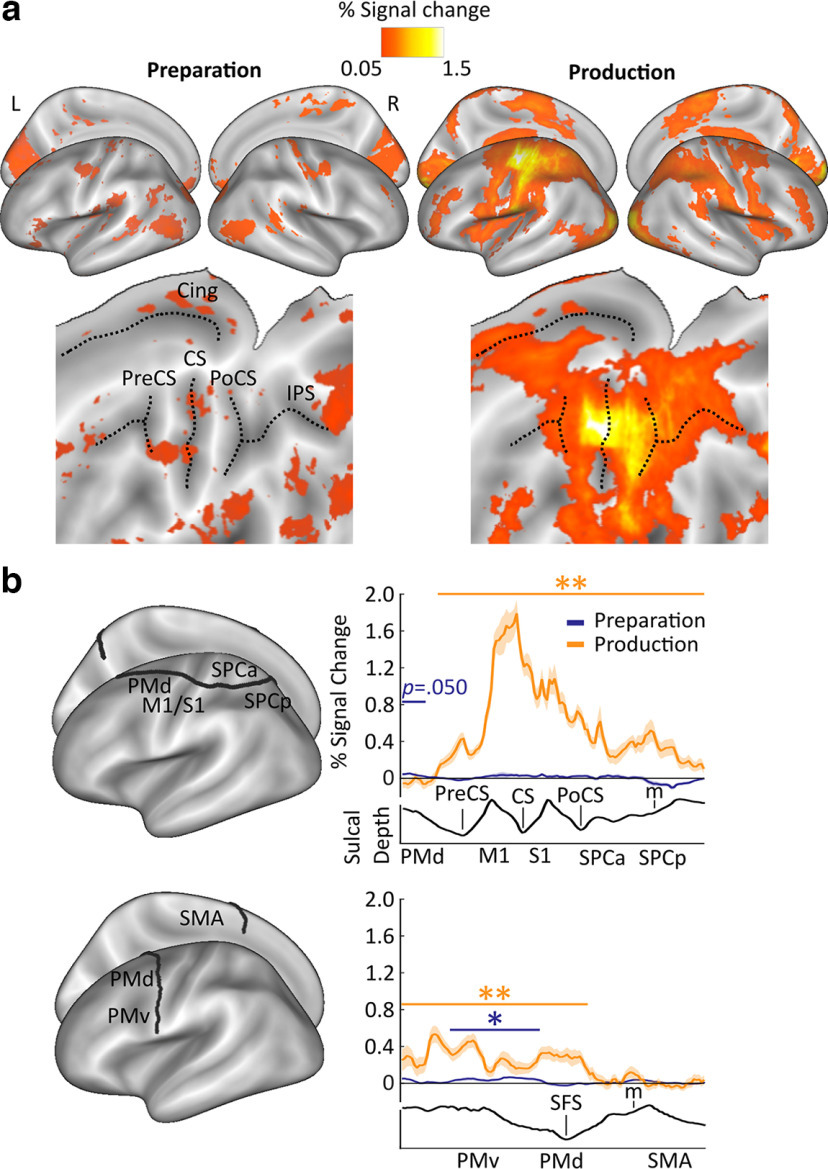
Percent signal change during preparation and production. ***a***, Inflated surface maps are shown in top panels and flat maps in bottom panels, displaying mean % signal change during preparation (left panels) and production relative to rest (right panels), respectively. For significant surface-based clusters across the cortex, see Table 2. ***b***, Mean % signal change relative to rest for both preparation (blue lines) and production (orange lines), plotted on cross-sections running from rostral premotor cortex, through the hand area, to the occipito-parietal junction (top) and on a profile running from the ventral, through the PMd, to the SMA (BA6; bottom). Clusters with increases above baseline are denoted by the colored horizontal lines and asterisks, calculated using one-tailed nonparametric permutation tests. Cing, Cingulate; CS, central sulcus; IPS, intraparietal sulcus; m, medial wall; OPJ, occipito-parietal junction; PoCS, postcentral sulcus; PreCS, precentral sulcus; SFS, superior frontal sulcus. **p < 0.01; *p < 0.05; one-sided *t* test. Shaded areas around the lines in b and c represent standard error of the sample mean.

Relative increases or decreases in the BOLD activity can be dissociated from the presence of informational content in an area, especially as efficiency increases and effort decreases with motor training (Wiestler and Diedrichsen, 2013; Berlot et al., 2020). Movement planning sometimes involves a decrease or no change relative to baseline in motor-related cortical areas while information about the upcoming action is still present in these regions (Gale et al., 2021; Ariani et al., 2022). However, planning can also involve increases in BOLD activity in premotor to parietal areas (Gallivan et al., 2011; Ariani et al., 2015; Nambu et al., 2015). To characterize the physiological response in a task that required rapid planning and production of finger sequences from long-term memory, the percent signal change during preparation and production relative to rest were calculated. Preparatory activity was solely sampled from No-Go trials for % signal change and multivariate pattern analyses to separate the BOLD activity related to sequence planning from production in a fast event-related design (see Materials and Methods). We then calculated the percent signal change across the cortex (Fig. 4*a*) and extracted values along two cross-sections of the cortical surface on the contralateral (left) side to the motor effector (Kornysheva and Diedrichsen, 2014) (Fig. 4*b*). These cross-sections extended from anterior to posterior and ventral to dorsal, across premotor to parietal and premotor to supplementary motor regions, respectively, because our hypotheses (www.osf.io/g64hv) on the imaging results during sequence preparation and production were put forward for contralateral premotor, primary motor, and parietal regions, which we expected to be tuned to sequence information based on previous studies (Tanji and Shima, 1994; Matsuzaka et al., 2007; Wymbs et al., 2012; Picard et al., 2013; Wiestler and Diedrichsen, 2013; Kornysheva and Diedrichsen, 2014; Wiestler et al., 2014; Yokoi et al., 2018; Berlot et al., 2020). We conducted one-tailed nonparametric permutation tests along these cross-sections to identify significant clusters where activity increased above baseline (Maris and Oostenveld, 2007). During preparation, a very small, but significant, activity increase was found within PMv (*p* = 0.002), and a marginally significant increase within PMd (*p* = 0.050; Fig. 4*b*), suggesting large variability across participants. During production, activity increases were found across the majority of contralateral motor-related regions, with one large cluster across PMd, M1, S1, SPCa, and SPCp (*p* < 0.001), and another cluster which spanned the cross-section from PMv to PMd (*p* < 0.001). The cross-section overlapping with anterior SMA did not show a significant activity increase from rest during production. However, note that the section of the SMA directly posterior to the cross-section did show a significant activity increase (for whole-brain contrast cluster analysis, Table 2).

Overall, no or small BOLD increases were observed across regions on the contralateral premotor-to-parietal axis during the short period of sequence planning. These results are in line with recent findings involving the motor planning of well-trained actions, for example, common object manipulations, such as grasping and lifting, or tapping with the same finger (Gale et al., 2021; Ariani et al., 2022).

### MVPA

**Figure 5.**
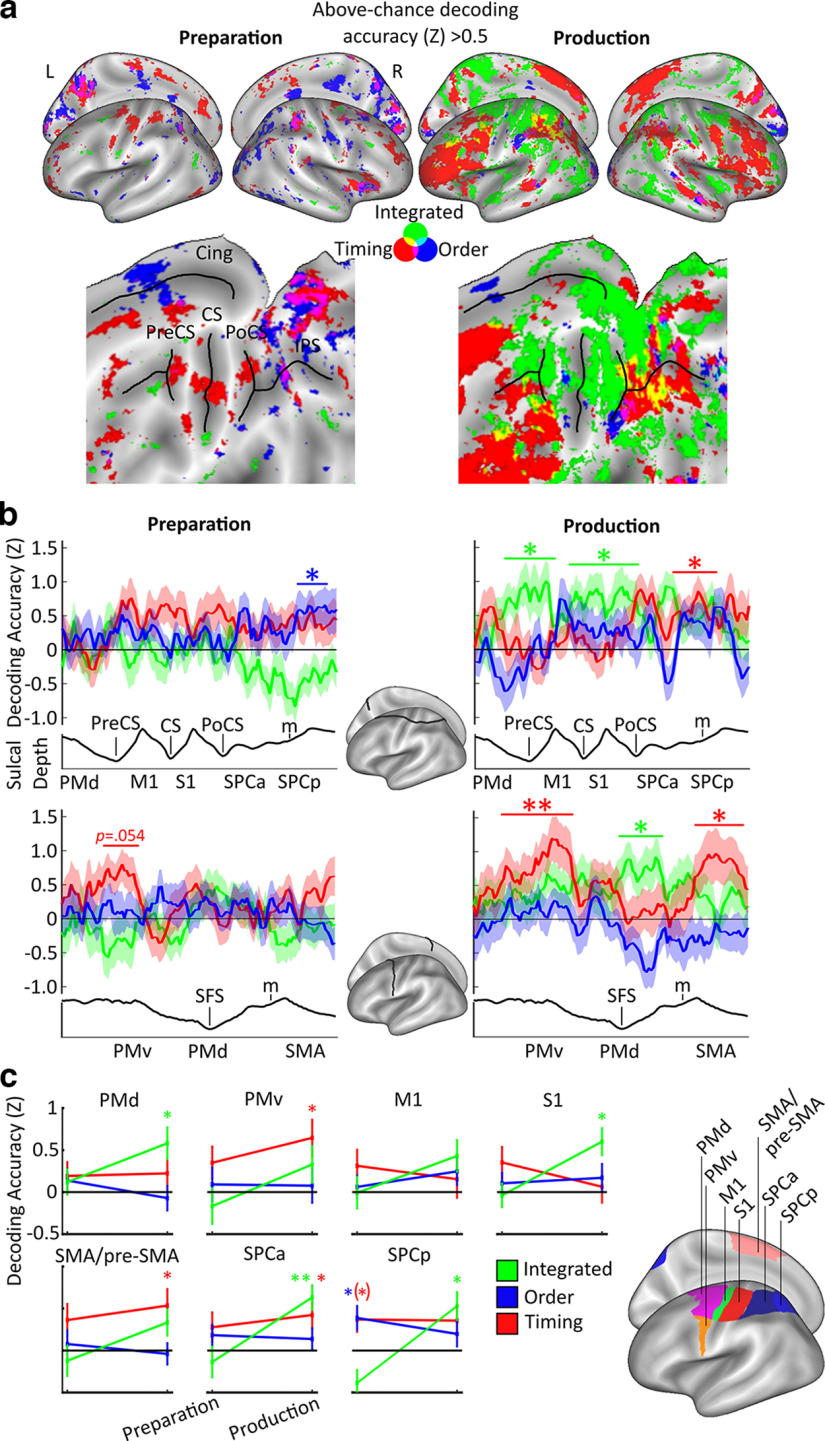
Multivariate pattern classification results. ***a***, Inflated surface (top panels) and flat maps (bottom panels), showing mean decoding *z*-accuracy values above chance for finger order (blue), timing (red), and integrated sequence patterns (green) (Kornysheva and Diedrichsen, 2014). For the corresponding significant surface-based clusters across the cortex, see Table 3. ***b***, Mean decoding *z*-accuracy values for each classifier along the cross-sections explained in Figure 4*b*. Colored asterisks indicate the respective above-chance clusters for each classifier during preparation and production. ***c***, Searchlight *z*-accuracy values were extracted using predetermined ROIs (Wiestler and Diedrichsen, 2013; Kornysheva and Diedrichsen, 2014; Yokoi et al., 2018) shown in the left panel. Colored asterisks indicate decoding above chance. (*)*p* = 0.060; **p < 0.01; *p < 0.05; one-sided *t* test above chance (Bonferroni-corrected for six comparisons within each ROI). Shaded areas around and error bars in b and c represent standard error of the sample mean.

We used MVPA to identify cortical areas that showed systematic changes in BOLD activity patterns between sequences with different finger orders, temporal structures, and unique combinations of the latter. Using a whole-brain searchlight of 160 voxels (Oosterhof et al., 2011), we trained a linear discriminant analysis classifier to distinguish between sequences in a one-run-out cross-validation method, an approach that has been validated with pattern simulations in a previous study (Kornysheva and Diedrichsen, 2014). Specifically, we looked for regional activity patterns that either transferred across or were unique for specific combinations of order and timing. The order classifier was used to decode between sequences with different finger orders, regardless of their pairing with a timing feature, whereas the timing classifier was trained to decode between sequences with different finger timings regardless of their pairing with a specific finger order. These two classifiers allowed the identification of regions which contained above-chance decoding of sequence order and timing independently of the other sequence feature, respectively (Fig. 5*a*). The integrated classifier decoded residual patterns after subtracting averaged sequence order and timing-related patterns for each run separately, to detect regions which hold information on sequence identity that is not driven by a simple summation of order and timing information (see Materials and Methods).

To reveal the continuous profile of feature decoding along contralateral motor regions on the cortical surface, we used the same permutation test approach (Maris and Oostenveld, 2007) as in the % signal change analysis for each of the three classifiers, for preparation and production, separately (Fig. 5*b*). During preparation, a significant cluster was found for finger order within SPCp (*p* = 0.040, *d* = 0.60), and a marginally significant cluster for timing decoding was identified within PMv (*p* = 0.054, *d* = 0.62). During production, above-chance decoding was shown for the integrated classifier within PMd in two clusters (*p* = 0.002, *d* = 0.81; *p* = 0.044, *d* = 0.63; on anterior to posterior and ventral to dorsal cross-sections, respectively) and S1, which extended into M1 and SPCa (*p* = 0.007, *d* = 0.79). Above-chance decoding of timing was found within SPCa (*p* = 0.016, *d* = 0.70), PMv (*p* < 0.001, *d* = 0.79), and SMA (*p* = 0.045, *d* = 0.53).

Next, we examined how well sequence features could be decoded from ROIs during preparation and production. These regions covered premotor to superior parietal areas: PMd, PMv, M1, S1, SMA/pre-SMA, SPCa, and SPCp. First, to identify above-chance decoding of sequence information in these areas, one-sample *t* tests were performed on the *z* values extracted from each of the predefined ROIs during both preparation and production for timing, order, and integrated classifiers (Fig. 5*c*). These *t* tests were Bonferroni-corrected 6 times, to account for phase (2) by classifier (3) within each predefined ROI. During preparation, the above chance was found in SPCp for sequence order decoding (*t*_(23)_ = 2.74, *p* = 0.036, *d* = 0.56), with marginally significant, but equal sized above-chance accuracy in SPCp for sequence timing (*t*_(23)_ = 2.51, *p* = 0.060, *d* = 0.51, Bonferroni-corrected). During production, classification increased above chance for sequence timing in SMA/pre-SMA (*t*_(23)_ = 2.71, *p* = 0.036, *d* = 0.56, Bonferroni-corrected), PMv (*t*_(23)_ = 3.00, *p* = 0.018, *d* = 0.61, Bonferroni-corrected), and SPCa (*t*_(23)_ = 2.67, *p* = 0.042, *d* = 0.55, Bonferroni-corrected). Further, classification increased above chance for order-timing integration in S1 (*t*_(23)_ = 3.69, *p* = 0.003, *d* = 0.75, Bonferroni-corrected), PMd (*t*_(23)_ = 3.06, *p* = 0.018, *d* = 0.63, Bonferroni-corrected), SPCa (*t*_(23)_ = 4.36, *p* < .001, *d* = 0.89, Bonferroni-corrected), and SPCp (*t*_(23)_ = 3.20, *p* = 0.012, *d* = 0.65, Bonferroni-corrected).

ROI analyses were only performed in the hemisphere contralateral to the movement in line with our hypotheses. For explorative purposes, we also conducted searchlight analyses across the whole cortex, including the ipsilateral surface, which were cluster- and Bonferroni-corrected for two hemispheres (Table 3). On the ipsilateral side, significant clusters during preparation were only found for order in the extrastriate visual cortex (*p* < 0.001, *p* = 0.004). During production, significant clusters were found for timing in M1 (*p* < 0.001), PMv (*p* < 0.001), inferior parietal (*p* > 0.001), and three clusters in lateral prefrontal (*p* > 0.001, *p* = 0.002, and *p* = 0.034) regions, with significant clusters for integration in inferior (*p* = 0.004) and superior parietal regions (*p* = 0.008). These findings suggest a general shift toward integration across phase across the cortex, with several regions also representing timing during production.

Finally, we set out to test our main hypotheses (Fig. 1, www.osf.io/g64hv) regarding an interaction between perimovement phase (preparation, production), classifier (timing, order, integrated), and region (PMd, PMv, M1, S1, SMA/pre-SMA, SPCa, SPCp). A repeated-measures ANOVA revealed a main effect of phase (*F*_(1,23)_ = 9.49, *p* = 0.005, η*p^2^* = 0.292), substantiating a general increase of decoding accuracy across regions and classifiers during production. The main effect of region was not significant (*F*_(3.84,88.42)_ = 0.45, *p* = 0.763, η*p^2^* = 0.019, Greenhouse-Geisser–corrected), suggesting that all the contralateral cortical ROIs had a comparable contribution to sequence decoding across trial phases. Importantly, we found a phase by classifier interaction (*F*_(2,46)_ = 10.34, *p* = 0.044, η*p^2^* = 0.127), which was driven by an overall increase in the integrated classifier accuracy from preparation (mean = −0.10, SE = 0.13) to production (mean = 0.49, SE = 0.11) (*p* = 0.003, 95% CI [0.217, 0.971], Bonferroni-corrected). Finally, we found no interaction of phase by region (*F*_(3.20,73.50)_ = 0.79, *p* = 0.512, η*p^2^* = 0.033, Greenhouse-Geisser–corrected), or phase by classifier by region (*F*_(5.40,124.18)_ = 1.63, *p* = 0.151, η*p^2^* = 0.066, Greenhouse-Geisser–corrected). In sum, this supports the hypothesis that tuning of these regions to high- and low-level features of sequences changes dynamically depending on trial phase, rather than region, with a state shift toward sequence feature integration after movement initiation across multiple regions.

## Discussion

Activity along the cortical premotor to parietal axis has been associated with motor sequence control, from its hierarchical organization (Gerloff et al., 1997; Sakai et al., 2003; Kennerley et al., 2004; Wiestler et al., 2014; Yokoi and Diedrichsen, 2019; Russo et al., 2020; Zimnik and Churchland, 2021) to sequence order and timing (Shima and Tanji, 1998; Ramnani and Passingham, 2001; Merchant et al., 2013; Crowe et al., 2014; Kornysheva and Diedrichsen, 2014; Wiestler et al., 2014). Yet how sequence-related computations in these regions unfold across planning and execution remains uncertain. Do these cortical areas retain a fixed tuning to sequence features and their integration throughout planning and execution? Or do they switch their content dynamically from before to after movement initiation? Here, we examined how motor cortical areas integrate informational content on the order of finger movement sequences and their timing across the planning and execution phases. Sequence decoding from activity patterns revealed that high-level features of sequence organization remain separate during movement planning and are integrated into unique patterns on movement initiation in premotor and parietal areas.

### Cortical patterns switch their tuning from planning to execution

**Figure 6.**
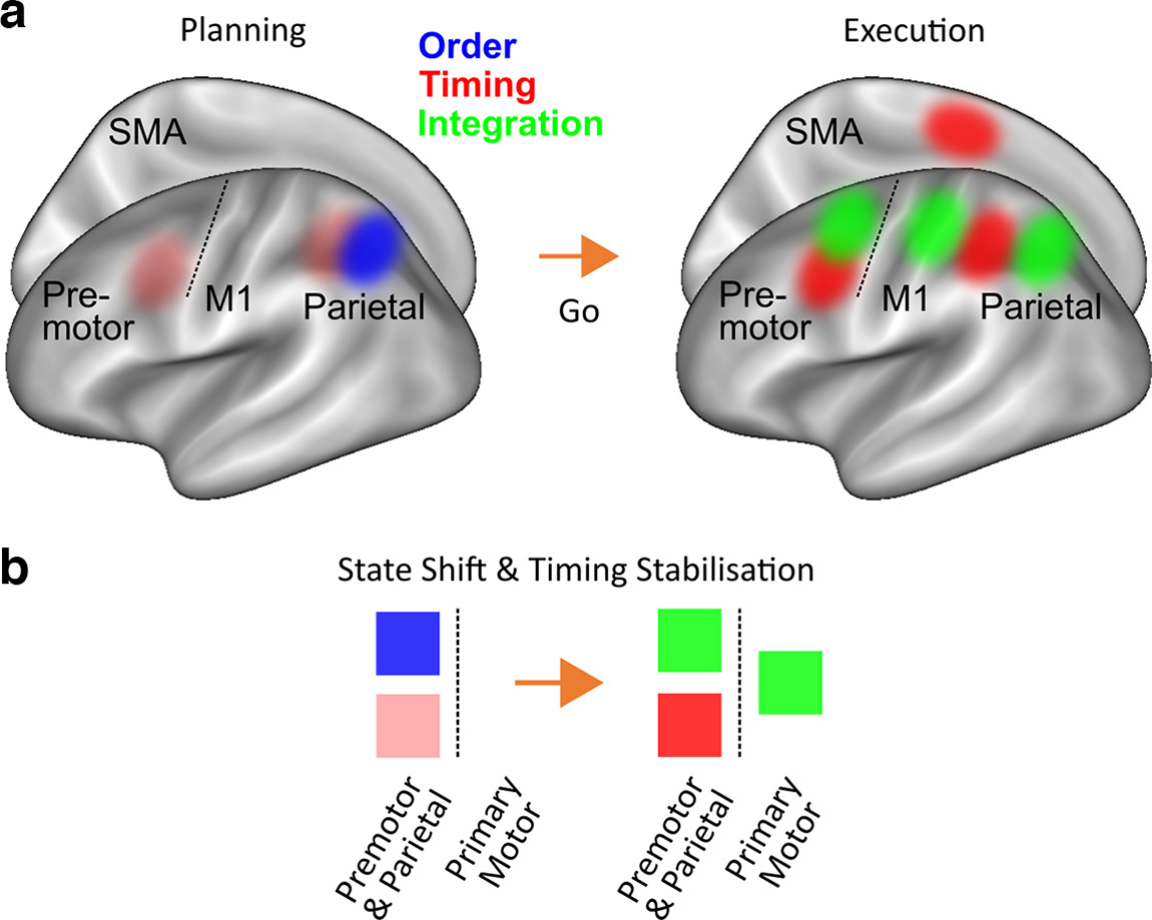
Schematic representation of sequence feature control during planning and integration across regions on the premotor-to-parietal axis contralateral to the movement. ***a***, Inflated cortical surface showing a schematic summary of the fMRI pattern decoding results. ***b***, These findings suggest that there is a shift within regions from planning to execution driven by the emergence of patterns related to the integration of sequence order and timing (Dynamic mapping: State shift). This is accompanied by pattern stabilization and increase within medial and lateral premotor and parietal areas related to timing (Fixed mapping: Higher-level stabilization). Notably, evidence for the control of movement order independently of its lower-level integration with timing is restricted to the planning phase only. Semi-transparent clusters of timing-related pattern decoding above chance reflect Bonferroni-corrected *p* values between 0.05 and 0.06 during planning.

Our results demonstrate a generalized dependency of cortical representations on perimovement phase, with a global shift across regions toward order and timing integration at the transition from sequence planning to execution. This indicates that most cortical motor-related areas do not rigidly map onto higher- versus lower-level representations of sequential organization, as assumed by earlier studies that focused on sequence execution alone (Diedrichsen et al., 2013; Kornysheva and Diedrichsen, 2014; Yokoi and Diedrichsen, 2019). Instead, pattern activity tuning in these regions changes dynamically on motor initiation (Fig. 6). Such a state switch in motor-related patterns echoes previous findings for the primary motor and dorsal premotor cortices in the context of single movements. These show that preparatory neural population activity occupies a different state space (“output-null”) from production to prevent readout from downstream areas during planning (Kaufman et al., 2014; O'Shea and Shenoy, 2016; Zimnik and Churchland, 2021). Here, cortical motor planning patterns are not simply subthreshold versions of execution activity patterns controlled by inhibitory gating within the cortex or downstream (Cisek and Kalaska, 2005; Duque and Ivry, 2009), but a qualitatively different neural activity pattern. Our results support the notion of a largely distinct functional tuning during motor planning across regions on the premotor to parietal axis in the context of sequential movements.

### Lack of sequence feature integration before motor initiation

As participants trained to perform the four finger sequences over 2 d and entirely from memory, one may expect that this level of practice would result in their retrieval as one integrated spatiotemporal synergy (Gentner et al., 2010). However, we found that information about motor sequence order and timing of the upcoming sequence was parsed trial-by-trial and integrated after motor initiation only. One possibility is that the 2 × 2 task design may encourage participants to hold onto higher-level representations of movement order and timing. However, in previous tasks where only one combination of order and timing was trained, transfer of known order and timing to new combinations were still found, showing that the separation is not dependent on the task structure but arises automatically (Ullén and Bengtsson, 2003; Kornysheva et al., 2013).

Although previous work has shown that planning-related activity in motor areas is predictive of movement features, such as speed, force, and trajectory of the upcoming movement (Pearce and Moran, 2012; Yang et al., 2015; Wong et al., 2016), these may be regarded as part of planning a holistic motor synergy (d'Avella et al., 2003; Shenoy et al., 2013; Overduin et al., 2015). In contrast, for discrete sequence learning, there is now ample evidence that higher-level sequence features, such as movement order and timing, are encoded independently (Ullén and Bengtsson, 2003; Bengtsson et al., 2004; Kornysheva and Diedrichsen, 2014; Zeid and Bullock, 2019) and remain so separate during planning (Kornysheva et al., 2019; Mantziara et al., 2021), despite training across multiple days and entirely memory-guided production. Specifically, sequence planning is do minated by higher-level control of motor sequences without precise implementation parameters (e.g., movement order without speed or timing information) (Mantziara et al., 2021), and ordinal position without effector information (Kornysheva et al., 2019). Further, the neural generation of sequence elements with a discrete timing goal (instructed delay or rapid succession) shows no fusion in M1, despite long-term training and fusion at the muscular level (Zimnik and Churchland, 2021). Yet, when and how integrated control is engaged during planning of more continuous overlapping movement sequences are uncertain. Rather than engaging a dedicated timing system, as is observed with discrete movements here and in previous work (Ullén and Bengtsson, 2003; Bengtsson et al., 2004; Medina et al., 2005; Kornysheva and Diedrichsen, 2014), continuously overlapping movements have been shown to use a state-dependent control system which integrates sensorimotor states of effectors (Ivry and Spencer, 2004; Diedrichsen et al., 2007; Ivry and Schlerf, 2008; Kornysheva, 2016). Thus, we predict that integrated control for continuous sequences would occur throughout planning and execution, unlike for discrete motor sequences.

What triggers sequence feature integration trial-by-trial? We propose that contralateral motor-related cortical regions activate movement order and timing plans separately until a sensory stimulus like the Go cue triggers the binding of the corresponding neural patterns. This binding may occur through subcortical, for example, thalamic input triggering an appropriate state for motor execution of specific combination of features (Wang et al., 2018; Inagaki et al., 2022). Delaying the binding of sequence features to the production phase and maintaining higher-level separation may allow the system to retain maximum flexibility trial-by-trial, should task demands change.

### Independent patterns for sequence timing, but not finger order, are reinstated during execution

We found a stark asymmetry between sequence order and timing during the sequence production phase. In contrast to the independent patterns for finger order, the activity patterns tuned to sequence timing increased (PMv) or emerged (SMA/pre-SMA, SPCa) during production. Thus, cortical patterns for sequence timing accompanied the emergence of sequence-specific integrated patterns, unlike patterns related to sequence order, which were restricted to the planning phase. This asymmetry was also observed at the behavioral level in the transfer task. Here, trained timing could be quickly recombined with a new order in line with previous work (Ullén and Bengtsson, 2003; Kornysheva et al., 2013, 2019; Kornysheva and Diedrichsen, 2014). In contrast, producing the same finger order with a new timing was associated with poorer performance, unlike a previous study involving a delayed sequence production from memory (Kornysheva et al., 2019). This interference effect suggests that participants were unable to separate the trained order from their timing during execution, which directly parallels the prominence of integrated and the lack of independent finger order tuning during motor production.

### M1 lacks information about sequences despite a large activity increase during execution

Our results show a lack of sequence feature separation or integration in contralateral M1 during preparation and only limited evidence for integration above chance during production extending out from the greater peak in S1. This occurs despite a large activity increase in M1 during production. While this contrasts with several older neuroimaging studies (Wiestler and Diedrichsen, 2013; Kornysheva and Diedrichsen, 2014; Nambu et al., 2015; Wymbs and Grafton, 2015), recent findings show that information held within M1 is not related to sequence control. Activity patterns in M1 do not change with sequence learning (Berlot et al., 2020, 2021) and reflect the processing of individual movements, particularly the first press of a sequence (Yokoi et al., 2018; Yokoi and Diedrichsen, 2019). Further, there has been no experimental evidence that sequential movements are neurally fused in M1 into holistic sequence representations: Constituent movements remain individuated in M1 regardless of sequential context (Russo et al., 2020; Zimnik and Churchland, 2021). Thus, matching the first finger press across trained sequences in each participant may explain why we see no prominent sequence feature decoding from M1 in contrast to a previous study on sequence timing and order (Kornysheva and Diedrichsen, 2014).

### Extending the motor planning framework to sequential actions

The framework for single movement motor planning proposes that the motor system enters a preparatory state that is distinct from movement execution (Churchland et al., 2010; Shenoy et al., 2013; Kaufman et al., 2014). Recent findings also suggest a distinction between the selection of motor goals and motor implementation planning, which formulate “what” movements to execute and “how” to execute them, respectively (Wong et al., 2015; Haith and Bestmann, 2020), converging with the idea of hierarchical motor sequence control (Diedrichsen and Kornysheva, 2015; Yokoi and Diedrichsen, 2019). Here, we propose that sequence order and timing features are specified during planning as “what” elements, representing higher-level control, and integrated during execution as “how” elements, representing lower-level implementation. Crucially, our results suggest that individual regions can undergo a state shift from “what” to “how” control depending on the perimovement phase. Future electrophysiological research should address whether the same neuronal populations are involved in both types of control within areas, and determine the neural origin and exact time point that triggers the informational state shift.
